# *ALOXE3* missense variant in a Chihuahua with autosomal recessive ichthyosis

**DOI:** 10.1111/age.70055

**Published:** 2025-10-07

**Authors:** Carina Vinberg, Stefan J. Rietmann, Sara Soto, Vidhya Jagannathan, Susanne Åhman, Tosso Leeb

**Affiliations:** ^1^ VetaDerm Veterinary Clinic Lund Sweden; ^2^ Institute of Genetics, Vetsuisse Faculty University of Bern Bern Switzerland; ^3^ Institute of Animal Pathology, Vetsuisse Faculty University of Bern Bern Switzerland

**Keywords:** Canis lupus familiaris, dermatology, dog, precision medicine, skin, whole genome sequencing

## Abstract

Ichthyoses are a heterogenous group of inherited disorders that are characterized by excessive scale formation on the skin. We investigated a Chihuahua with severe scaling since age 12 weeks. The scaling was generalized and involved the entire body and legs. The paw pads were mildly hyperkeratotic. The clinical features together with histopathological findings in skin biopsies were compatible with non‐epidermolytic ichthyosis. To identify a potential genetic cause of the ichthyosis, we sequenced the genome of the affected dog and compared the data to 1567 control genomes. Filtering for private variants identified a homozygous missense variant in *ALOXE3*, XP_038392720.1:p.(Gly460Asp). *ALOXE3* is a known candidate gene for ichthyosis in humans and encodes arachidonate epidermal lipoxygenase 3. The enzyme is involved in the production of a functional corneocyte lipid envelope, an essential component of the epidermal barrier. Pathogenic variants in *ALOXE3* have been described in human patients with autosomal recessive congenital ichthyosis. We assume that the identified missense variant in the affected Chihuahua of this study impairs the normal function of the ALOXE3 protein and the formation of a functioning corneocyte lipid envelope, which ultimately leads to a disorder of cornification that manifests as ichthyosis. To the best of our knowledge, this is the first report of a spontaneous *ALOXE3* variant in domestic animals.

## INTRODUCTION

Ichthyoses represent a heterogeneous group of heritable skin diseases characterized by generalized scaly and hyperkeratotic skin (Oji et al., 2010). In humans, at least 69 genes have been associated with different forms of ichthyosis, grouped into non‐syndromic ichthyoses, in which the clinical phenotype is exclusively limited to scaly skin, and syndromic ichthyoses involving additional organs (Fischer, 2009; Gutiérrez‐Cerrajero et al., 2023; Uitto et al., 2020). Further subdivision into epidermolytic and non‐epidermolytic ichthyoses is based on the presence or absence of light microscopic findings of vacuoles and lysis of keratinocytes (Mauldin, 2013). In dogs, several inherited ichthyoses have been described in different breeds. Currently, causal variants in nine different genes have been reported (Affolter et al., 2022; Bauer et al., 2017; Casal et al., 2017; Credille et al., 2005, 2009; Grall et al., 2012; Kiener et al., 2022; Kiener, Castilla, et al., 2023; Metzger et al., 2015). Functional candidate genes for ichthyoses are mainly involved in the biosynthesis, metabolism, and transport of lipids required for skin barrier function or the intracellular protein network responsible for the integrity of skin structure (Gutiérrez‐Cerrajero et al., 2023).

In Chihuahuas, so far, two single cases with clinically and genetically distinct forms of inherited ichthyoses have been reported. A homozygous *SDR9C7*:p.Arg152Trp missense variant was found in a Chihuahua with recessive non‐epidermolytic ichthyosis (Kiener, Castilla, et al., 2023). A heterozygous *KRT10*:p.Arg146His missense variant was identified in a Chihuahua with dominant epidermolytic hyperkeratosis. While the latter phenotype belongs to the large group of ichthyoses, the published dog was clinically mainly characterized by paw pad hyperkeratosis and had very little scale formation (Kiener, Åhman, et al., 2023).

The present study was prompted by a Chihuahua showing excessive scale formation on the skin. It did not carry any of the known pathogenic alleles in *KRT10* or *SDR9C7* (Kiener, Åhman, et al., 2023; Kiener, Castilla, et al., 2023). We therefore investigated the clinical and histopathological phenotype of this presumably new form of canine ichthyosis and initiated a genetic analysis to unravel the underlying molecular etiology.

## METHODS

### Animal selection

This study included samples from 43 Chihuahuas from the Vetsuisse Biobank (Table S1). Three of them were affected by ichthyoses or related cornification defects including the index case of this study and two other ichthyotic Chihuahuas that had been characterized in detail in earlier studies from our group. One of them was homozygous mutant at the previously reported *SDR9C7* variant (Kiener, Castilla, et al., 2023). The other was affected with a previously reported *KRT10*‐related dominant form of ichthyosis (Kiener, Åhman, et al., 2023). All relevant genotypes of these dogs are given in Table S1.

### Clinical and histopathological examinations

The affected Chihuahua was examined by a resident and a board‐certified specialist for veterinary dermatology due to progressive and generalized scaling. Three formalin‐fixed haired skin biopsies were taken from the dog, routinely processed in paraffin, sectioned and stained with hematoxylin and eosin. Subsequent histopathological examination was performed by a board‐certified specialist for veterinary pathology.

### DNA extraction and whole genome sequencing

Genomic DNA was extracted from EDTA blood samples using a Maxwell RSC Whole Blood Kit on a Maxwell RSC instrument (Promega, Dübendorf, Switzerland). For whole genome sequencing of the affected dog, a PCR‐free library with an insert size of approximately 400 bp was prepared. Sequencing was performed with 2 × 150‐bp reads on an Illumina NovaSeq 6000 instrument at 21.8× coverage (Illumina, Zürich, Switzerland). Reads were mapped to the UU_Cfam_GSD_1.0 reference genome assembly as described (Jagannathan et al., 2019).

### Variant calling and filtering

The GATK Haplotype Caller was used in gVCF mode to call single nucleotide and small indel variants according to a previously described workflow (Jagannathan et al., 2019; McKenna et al., 2010). To predict the functional effects of the identified variants, the snpeff software was used in combination with the NCBI annotation release 106 (Cingolani et al., 2012). To detect private variants, the whole genome sequencing data of the index case was filtered against 1567 control dogs from various breeds. Variants with a snpeff predicted effect of “high” or “moderate” were considered protein changing. Accession numbers of all used genome sequences are listed in Table S2.

### PCR amplification and Sanger sequencing

For targeted genotyping of the *ALOXE3*:XM_038536792.1:c.1379G>A missense variant, a primer pair for the amplification of a 525‐bp product containing the variant was designed. PCR amplification on genomic DNA samples using primers 5′‐GAAGGGACGGTCCAGCTC‐3′ and 5′‐GGTGGTGGCCCTACTATGTG‐3′ was performed using AmpliTaqGold360Mastermix (Thermo Fisher Scientific, Waltham, MA, USA). PCR products were purified using exonuclease I and alkaline phosphatase.

Subsequent Sanger sequencing was done on an ABI 3730 DNA analyzer in two separate reactions using the forward or reverse primer with an ABI BigDye v3.1 sequencing kit (Thermo Fisher Scientific, Waltham, MA, USA). The resulting data were analyzed using the sequencher 5.1 software (GeneCodes, Ann Arbor, MI, USA).

### In silico protein analyses

We modeled the three‐dimensional structure of the wildtype and mutant canine ALOXE3 protein with Colabfold (Mirdita et al., 2022). Pathogenicity predictions for the XP_038392720.1:p.Gly460Asp variant were performed with the programs PredictSNP, SNPs&Go and MutPred2 (Bendl et al., 2014; Calabrese et al., 2009; Pejaver et al., 2020).

## RESULTS

### Clinical examination

A 5‐month‐old male Chihuahua had been referred due to generalized scaling, progressively worsening since age 12 weeks. In addition, he had moderate ear pruritus. Topical treatment with Advocate® Spot‐On Solution (Imidacloprid, Moxidectin) and Betamethasone ear drops had not improved clinical signs.

**FIGURE 1:**
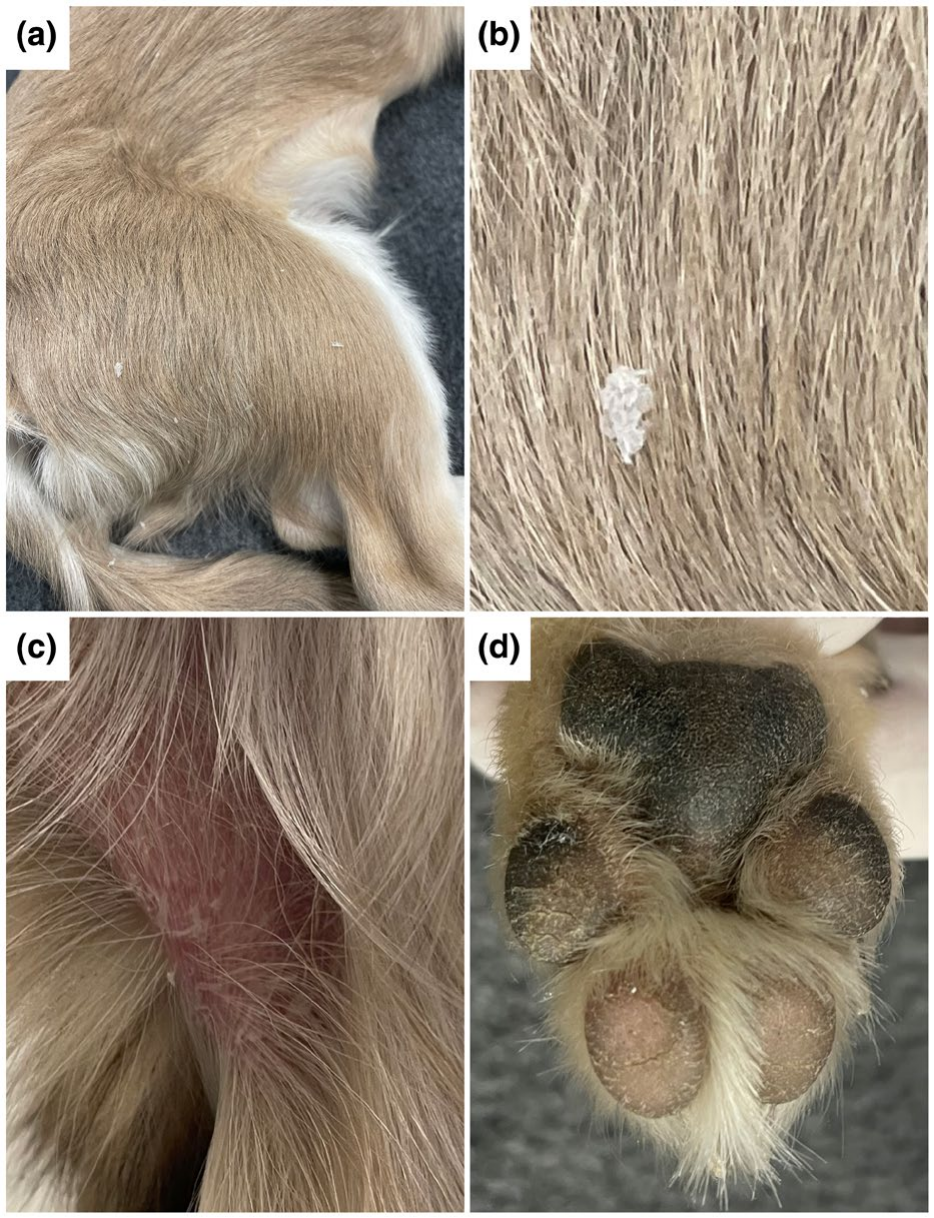
Clinical phenotype of the affected Chihuahua. (a) Diffuse large white scale throughout the haircoat. (b) Magnification of a scale. (c) Scaly and erythematous skin in the inguinal region. (d) Mildly hyperkeratotic paw pads with minor fissures.

At presentation, the dog had excessive scaling affecting the entire body except the head. Large, white scales (1–5 mm) were widespread, many with embedded hair shafts. The paw pads on all feet were mildly hyperkeratotic (Figure 1). The pinnae were normal, but the ear canals were covered with a thick layer of dry white‐to‐yellow scales. Clinical examination in other aspects was unremarkable except for a prominent mandibular malocclusion, prognathism, a feature not uncommon in this breed.

As a part of the diagnostic work‐up, three punch biopsies were taken under sedation and local anesthesia from the skin of the dorsal shoulder area and dorsal thoraco‐lumbar area for histopathological evaluation.

The dog was followed until age 1.5 years and was mainly treated with topical therapy once weekly alternating keratolytic shampoo to remove excessive scale and a moisturizing shampoo, which improved the condition somewhat. Ear canals were cleaned once weekly and treated with ear drops containing olive and mineral oil (Vaxol®) to remove scales building up in the ear canals. To relieve ear pruritus, topical hydrocortisone solution (Locoid®) was used. Furthermore, from age 6 months the dog gradually developed clinical signs of allergic skin disease in addition to the ichthyosis. It showed increasing, moderate pruritus involving the paws and inguinal areas and was gradually becoming hyperpigmented in these areas. The pruritus responded well to treatment with subcutaneous lokivetmab (3 mg/kg, Cytopoint, Zoetis).

### Histopathological examination

**FIGURE 2:**
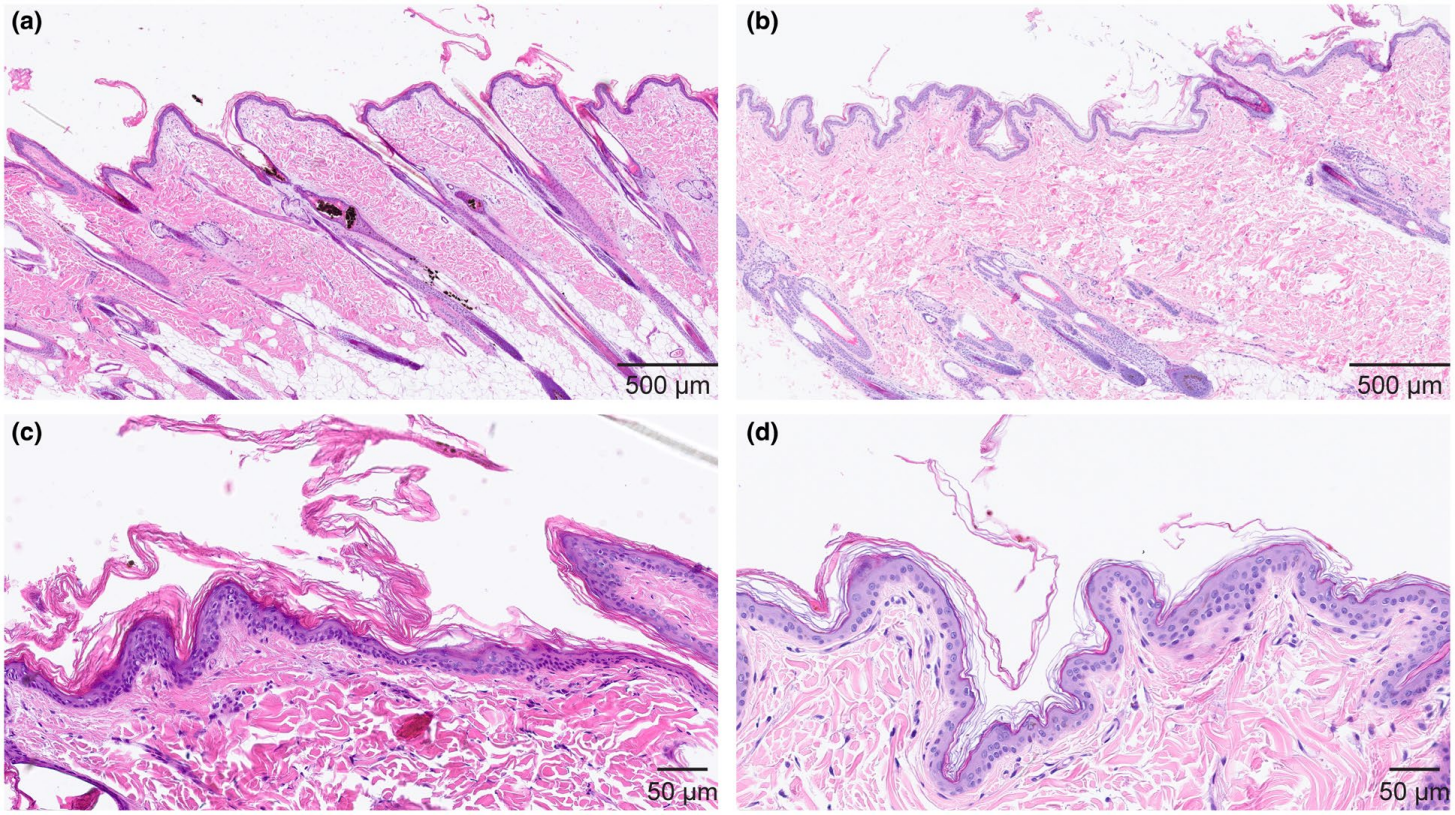
Histopathological phenotype of the affected Chihuahua. (a, b) Overview at low magnification. (c, d) Higher magnifications. (a, c) Skin punch biopsy of the affected Chihuahua at age 5 months presenting on its surface a markedly thickened stratum corneum displaying increased amount of orthokeratotic keratin of lamellar type, compatible with a non‐epidermolytic ichthyosis (hematoxylin and eosin stain). (b, d) Control skin biopsy of a 1‐year‐old dog presenting an unremarkable stratum corneum, both in thickness and type of keratin (basket‐weave; hematoxylin and eosin stain).

Findings were similar in all three biopsies, characterized by markedly increased amounts of keratin in the stratum corneum (marked lamellar orthokeratotic hyperkeratosis), with keratin lifting and flaking, explaining the clinical scaling (Figure 2). Mildly increased amounts of keratin in the infundibular hair follicle lumen were also observed (infundibular hyperkeratosis). The rest of the cutaneous structures, including stratum granulosum and dermis, were unremarkable. The histopathological features together with the anamnesis were interpreted as non‐epidermolytic ichthyosis.

### Genetic analysis

As an inherited disease was suspected, the genome of the affected Chihuahua was sequenced, and the data were compared to 1567 control genomes. Filtering for private variants in the affected dog identified 63 private protein changing variants, of which only one resided in a known functional candidate gene for ichthyosis (Table 1, Tables S3 and S4).

**TABLE 1:** Results of variant filtering in the affected Chihuahua against 1567 control genomes.

Filtering step	Heterozygous variants	Homozygous variants
All variants	3 351 192	2 723 532
Private variants	4814	889
Private protein changing variants	56	7
Private protein changing variants in 21 functional candidate genes	0	1

**FIGURE 3:**
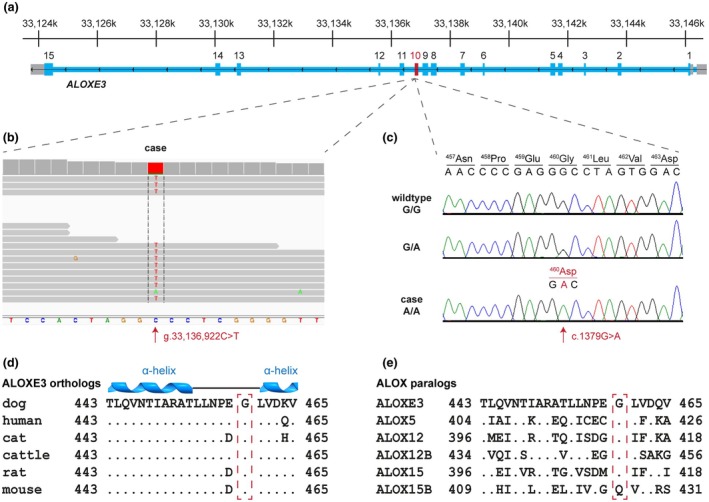
Details of the *ALOXE3*:p.(Gly460Asp) variant identified in the affected Chihuahua. (a) Genomic organization of the *ALOXE3* gene on chromosome 5. Coordinates refer to the UU_Cfam_GSD1.0 reference assembly. The exon containing the variant is highlighted in red. (b) Integrative genomics viewer (IGV) screenshot showing the missense variant (Robinson et al., 2011). Note that the *ALOXE3* gene is in reverse complementary orientation with respect to the genome reference sequence. (c) Sanger sequencing results of three Chihuahuas, representing the different *ALOXE3* genotypes in the orientation of transcription. (d) Partial amino acid alignment of the ALOXE3 protein in different mammalian species. The protein sequence including glycine‐480 is highly conserved between all depicted species. Predicted α‐helical secondary structure elements are indicated. (e) Partial amino acid alignment of the six human ALOX paralogs, of which five share the conserved glycine.

The identified candidate causal variant was a homozygous single nucleotide substitution affecting the *ALOXE3* gene, NC_049226.1:g.33136922C>T or XM_038536792.1:c.1379G>A. It is a missense variant, XP_038392720.1:p.(Gly460Asp), predicted to exchange an evolutionarily conserved glycine with an aspartic acid (Figure 3).

The ALOXE3 protein is highly conserved across mammalian species and consists of 711 amino acids in humans and dogs, of which 630 (89%) are identical. The protein is composed of an N‐terminal PLAT domain (~aa 1–119) and a catalytic C‐terminal lipoxygenase homology domain (~aa 120–711). No experimental three‐dimensional structure of a mammalian ALOXE3 has been reported. However, Alphafold‐based models of the structure show high similarities to the experimentally solved structure of the homologous ALOX15 (Gillmor et al., 1997). We compared Alphafold‐based models of the canine wildtype and Gly460Asp mutant proteins. The amino acid substitution was not predicted to cause a major alteration of the backbone structure. However, it is conceivable that the introduction of a relatively large and negatively charged side chain might interfere with binding of the hydrophobic ligand or another important aspect of ALOXE3 function. Three different programs predicted the Gly460Asp missense variant to be pathogenic or deleterious (PredictSNP probability 87%; SNPs&Go probability 0.845; MutPred2 score 0.935).

The *ALOXE3*:p.Gly460Asp missense variant was subsequently genotyped by Sanger sequencing in the entire cohort of 43 Chihuahuas. The index case was confirmed to be homozygous for the mutant allele (Figure 3c). One unaffected dog without known relations to the index case was heterozygous and the other 41 dogs were homozygous for the wildtype allele (Table S1).

## DISCUSSION

In this study, we investigated a young Chihuahua dog presenting with generalized scaling from age 12 weeks, suspected to suffer from hereditary ichthyosis. Histopathological lesions were typical of non‐epidermolytic ichthyosis (Mauldin, 2013). Whole genome sequencing of the affected dog revealed a missense variant in *ALOXE3*, a well‐known candidate gene for autosomal recessive congenital ichthyosis in humans (Gutiérrez‐Cerrajero et al., 2023).

The dog subsequently developed atopic dermatitis. This is consistent with earlier observations that ichthyoses lead to skin barrier defects and therefore constitute an important predisposing risk factor for developing atopic disease (Elias, 2018). Recurrent atopic eczema was also reported in a human patient with *ALOXE3*‐related ichthyosis (Takeichi et al., 2017).

The identified genetic variant in *ALOXE3* suggests deficiency of a lipoxygenase as underlying cause of the ichthyosis in the affected Chihuahua. Lipoxygenases are a group of lipid‐peroxidizing enzymes present in animals, plants and fungi. Mammals have six genes encoding different lipoxygenases (Zhuravlev et al., 2024). The lipoxygenases encoded by *ALOXE3* and *ALOX12B* in particular are essential in the formation of the epidermal barrier (Muñoz‐Garcia et al., 2013). In contrast to other members of the enzyme family, ALOXE3 primarily exhibits a hydroperoxide isomerase activity (Zheng et al., 2011). It acts in sequence with ALOX12B to process essential fatty acid derived substrates into epoxy‐keton derivatives which are involved in the conjugation of ω‐hydroxyceramide to membrane proteins. Thereby ALOX12B and ALOXE3 contribute to the formation of the corneocyte lipid envelope (Krieg & Fürstenberger, 2014; Zheng et al., 2011). In combination with the cornified cell envelope and intercellular lipid layers, the corneocyte lipid envelope is a key element of the cornified epidermal layers and crucial for maintaining a functional cutaneous barrier (Akiyama, 2021).

The identified ALOXE3:p.Gly460Asp variant is predicted to affect the lipoxygenase domain of the encoded protein. As a non‐polar glycine is replaced by a much larger and negatively charged aspartate, the protein function might be impaired. This is also supported by three independent in silico pathogenicity prediction tools.

Multiple pathogenic *ALOXE3* variants were reported in human patients with autosomal recessive ichthyosis 3. They commonly involve a rather mild to moderate phenotype similar to the investigated Chihuahua (Eckl et al., 2005, 2009; Hotz et al., 2021; Takeichi et al., 2017). Missense variants are the second most common pathogenic alterations in the *ALOXE3* gene in humans and mostly occur in exon 4–16 encoding the lipoxygenase domain. While multiple pathogenic missense variants affecting the lipoxygenase domain have been characterized, a variant homologous to the canine p.Gly460Asp has not yet been reported in human patients.

*Aloxe3* ^*−/−*^ knockout mice showed abnormalities in the skin structure and development, including a tightly packed stratum corneum, hyperkeratosis and a significant decrease of protein‐bound ω‐hydroxyceramides. Most of them were reported to die soon after birth, presumably due to impairment of the skin barrier and resulting transepidermal water loss (Krieg et al., 2013). To the best of our knowledge, no spontaneously occurring *ALOXE3* associated ichthyosis in an animal has been reported so far.

While we consider the *ALOXE3*:p.(Gly460Asp) variant as likely to be causal for the observed ichthyosis in the affected Chihuahua, we acknowledge limitations of our investigation, which is based on a single isolated case. We were therefore not able to compile sufficient evidence to classify this variant as pathogenic or likely pathogenic according to the human ACMG/AMP criteria (Richards et al., 2015). According to ACMG/AMP criteria it would therefore have to be formally classified as variant of uncertain significance. Our main arguments for the causality of the *ALOXE3* variant are: (i) *ALOXE3* is a well characterized functional candidate gene for a highly characteristic comparable phenotype in humans; (ii) the in silico predicted damaging effect of the missense variant on the ALOXE3 protein; and (iii) absence of the homozygous mutant genotype from healthy controls and overall a very low frequency of the mutant allele in dogs.

In our control cohort we discovered an unaffected Chihuahua carrying the mutant *ALOXE3* allele in a heterozygous state. This dog did not have any known relation to the affected dog and originated in another country. This might indicate that the mutant allele may have already spread in the population.

In conclusion, to the best of our knowledge, this study provides the first report of a probably *ALOXE3* associated ichthyosis in a domestic animal. The identification of a plausible candidate causal variant enables genetic testing of dogs to prevent unintentional carrier × carrier matings and the breeding of further affected dogs.

## AUTHOR CONTRIBUTIONS

**Carina Vinberg:** Investigation; visualization; writing – original draft; writing – review and editing. **Stefan J. Rietmann:** Investigation; visualization; writing – original draft; writing – review and editing. **Sara Soto:** Investigation; visualization; writing – original draft; writing – review and editing. **Vidhya Jagannathan:** Data curation; writing – review and editing. **Susanne Åhman:** Conceptualization; supervision; writing – original draft; writing – review and editing. **Tosso Leeb:** Conceptualization; visualization; writing – original draft; writing – review and editing; supervision.

## FUNDING INFORMATION

This research was funded by the Swiss National Science Foundation, grant number 310030_200354.

## CONFLICT OF INTEREST STATEMENT

The authors declare no conflict of interest.

## ETHICS STATEMENT

The affected dogs in this study were privately owned and blood samples for diagnostic purposes were collected with the consent of their owners. The collection of blood samples from healthy control dogs was approved by the “Cantonal Committee for Animal Experiments” (Canton of Bern; permit BE94/2022).

## Supporting information


Table S1.

Table S2.

Table S3.

Table S4.

## Data Availability

The genome sequence data were submitted to the European Nucleotide Archive (ENA). All accession numbers are listed in Table S1.

## References

[age70055-bib-0001] Affolter, V.K. , Kiener, S. , Jagannathan, V. , Nagle, T. & Leeb, T. (2022) A de novo variant in the keratin 1 gene (KRT1) in a Chinese shar‐pei dog with severe congenital cornification disorder and non‐epidermolytic ichthyosis. PLoS One, 17(10), e0275367. Available from: 10.1371/journal.pone.0275367 36251712 PMC9576078

[age70055-bib-0002] Akiyama, M. (2021) Acylceramide is a key player in skin barrier function: insight into the molecular mechanisms of skin barrier formation and ichthyosis pathogenesis. FEBS Journal, 288(7), 2119–2130. Available from: 10.1111/febs.15497

[age70055-bib-0003] Bauer, A. , Waluk, D.P. , Galichet, A. , Timm, K. , Jagannathan, V. , Sayar, B.S. et al. (2017) A de novo variant in the *ASPRV1* gene in a dog with ichthyosis. PLoS Genetics, 13(3), e1006651. Available from: 10.1371/journal.pgen.1006651 28249031 PMC5352138

[age70055-bib-0004] Bendl, J. , Stourac, J. , Salanda, O. , Pavelka, A. , Wieben, E.D. , Zendulka, J. et al. (2014) PredictSNP: robust and accurate consensus classifier for prediction of disease‐related mutations. PLoS Computational Biology, 10(1), e1003440. Available from: 10.1371/journal.pcbi.1003440 24453961 PMC3894168

[age70055-bib-0005] Calabrese, R. , Capriotti, E. , Fariselli, P. , Martelli, P.L. & Casadio, R. (2009) Functional annotations improve the predictive score of human disease‐related mutations in proteins. Human Mutation, 30(8), 1237–1244. Available from: 10.1002/humu.21047 19514061

[age70055-bib-0006] Casal, M.L. , Wang, P. , Mauldin, E.A. , Lin, G. & Henthorn, P.S. (2017) A defect in NIPAL4 is associated with autosomal recessive congenital ichthyosis in American bulldogs. PLoS One, 12(1), e0170708. Available from: 10.1371/journal.pone.0170708 28122049 PMC5266318

[age70055-bib-0007] Cingolani, P. , Platts, A. , Wang, L.L. , Coon, M. , Nguyen, T. , Wang, L. et al. (2012) A program for annotating and predicting the effects of single nucleotide polymorphisms, SnpEff: SNPs in the genome of *Drosophila melanogaster* strain w1118; iso‐2; iso‐3. Fly, 6(2), 80–92. Available from: 10.4161/fly.19695 22728672 PMC3679285

[age70055-bib-0008] Credille, K.M. , Barnhart, K.F. , Minor, J.S. & Dunstan, R.W. (2005) Mild recessive epidermolytic hyperkeratosis associated with a novel keratin 10 donor splice‐site mutation in a family of Norfolk terrier dogs. British Journal of Dermatology, 153(1), 51–58. Available from: 10.1111/j.1365-2133.2005.06735.x 16029326

[age70055-bib-0009] Credille, K.M. , Minor, J.S. , Barnhart, K.F. , Lee, E. , Cox, M.L. , Tucker, K.A. et al. (2009) Transglutaminase 1‐deficient recessive lamellar ichthyosis associated with a LINE‐1 insertion in Jack Russell terrier dogs. British Journal of Dermatology, 161(2), 265–272. Available from: 10.1111/j.1365-2133.2009.09161.x 19438474

[age70055-bib-0010] Eckl, K.‐M. , de Juanes, S. , Kurtenbach, J. , Nätebus, M. , Lugassy, J. , Oji, V. et al. (2009) Molecular analysis of 250 patients with autosomal recessive congenital ichthyosis: evidence for mutation hotspots in *ALOXE3* and allelic heterogeneity in *ALOX12B* . Journal of Investigative Dermatology, 129(6), 1421–1428. Available from: 10.1038/jid.2008.409 19131948

[age70055-bib-0011] Eckl, K.‐M. , Krieg, P. , Küster, W. , Traupe, H. , André, F. , Wittstruck, N. et al. (2005) Mutation spectrum and functional analysis of epidermis‐type lipoxygenases in patients with autosomal recessive congenital ichthyosis. Human Mutation, 26(4), 351–361. Available from: 10.1002/humu.20236 16116617

[age70055-bib-0012] Elias, P.M. (2018) Primary role of barrier dysfunction in the pathogenesis of atopic dermatitis. Experimental Dermatology, 27(8), 847–851. Available from: 10.1111/exd.13693 29799646

[age70055-bib-0013] Fischer, J. (2009) Autosomal recessive congenital ichthyosis. Journal of Investigative Dermatology, 129(6), 1319–1321. Available from: 10.1038/jid.2009.57 19434086

[age70055-bib-0014] Gillmor, S.A. , Villaseñor, A. , Fletterick, R. , Sigal, E. & Browner, M.F. (1997) The structure of mammalian 15‐lipoxygenase reveals similarity to the lipases and the determinants of substrate specificity. Nature Structural Biology, 4(12), 1003–1009. Available from: 10.1038/nsb1297-1003 9406550

[age70055-bib-0015] Grall, A. , Guaguère, E. , Planchais, S. , Grond, S. , Bourrat, E. , Hausser, I. et al. (2012) PNPLA1 mutations cause autosomal recessive congenital ichthyosis in golden retriever dogs and humans. Nature Genetics, 44(2), 140–147. Available from: 10.1038/ng.1056 22246504

[age70055-bib-0016] Gutiérrez‐Cerrajero, C. , Sprecher, E. , Paller, A.S. , Akiyama, M. , Mazereeuw‐Hautier, J. , Hernández‐Martín, A. et al. (2023) Ichthyosis. Nature Reviews Disease Primers, 9(1), 2. Available from: 10.1038/s41572-022-00412-3 36658199

[age70055-bib-0018] Hotz, A. , Kopp, J. , Bourrat, E. , Oji, V. , Komlosi, K. , Giehl, K. et al. (2021) Meta‐analysis of mutations in *ALOX12B* or *ALOXE3* identified in a large cohort of 224 patients. Genes, 12(1), 80. Available from: 10.3390/genes12010080 33435499 PMC7826849

[age70055-bib-0019] Jagannathan, V. , Drögemüller, C. , Leeb, T. , Aguirre, G. , André, C. , Bannasch, D. et al. (2019) A comprehensive biomedical variant catalogue based on whole genome sequences of 582 dogs and eight wolves. Animal Genetics, 50(6), 695–704. Available from: 10.1111/age.12834 31486122 PMC6842318

[age70055-bib-0020] Kiener, S. , Åhman, S. , Jagannathan, V. , Soto, S. & Leeb, T. (2023) Heterozygous *KRT10* missense variant in a Chihuahua with severe epidermolytic ichthyosis. Animal Genetics, 54(5), 652–654. Available from: 10.1111/age.13341 37332248

[age70055-bib-0021] Kiener, S. , Castilla, E. , Jagannathan, V. , Welle, M. & Leeb, T. (2023) *SDR9C7* missense variant in a Chihuahua with non‐epidermolytic ichthyosis. Animal Genetics, 54(4), 562–565. Available from: 10.1111/age.13319 36967672

[age70055-bib-0022] Kiener, S. , Wiener, D.J. , Hopke, K. , Diesel, A.B. , Jagannathan, V. , Mauldin, E.A. et al. (2022) *ABHD5* frameshift deletion in golden retrievers with ichthyosis. G3: Genes, Genomes, Genetics, 12, jkab397. Available from: 10.1093/g3journal/jkab397 34791225 PMC9210301

[age70055-bib-0023] Krieg, P. & Fürstenberger, G. (2014) The role of lipoxygenases in epidermis. Biochimica et Biophysica Acta—Molecular and Cell Biology of Lipids, 1841(3), 390–400. Available from: 10.1016/j.bbalip.2013.08.005 23954555

[age70055-bib-0024] Krieg, P. , Rosenberger, S. , De Juanes, S. , Latzko, S. , Hou, J. , Dick, A. et al. (2013) Aloxe3 knockout mice reveal a function of epidermal lipoxygenase‐3 as hepoxilin synthase and its pivotal role in barrier formation. Journal of Investigative Dermatology, 133(1), 172–180. Available from: 10.1038/jid.2012.250 22832496

[age70055-bib-0025] Mauldin, E.A. (2013) Canine ichthyosis and related disorders of cornification. Veterinary Clinics of North America: Small Animal Practice, 43(1), 89–97. Available from: 10.1016/j.cvsm.2012.09.005 23182326 PMC3529142

[age70055-bib-0026] McKenna, A. , Hanna, M. , Banks, E. , Sivachenko, A. , Cibulskis, K. , Kernytsky, A. et al. (2010) The genome analysis toolkit: a MapReduce framework for analyzing next‐generation DNA sequencing data. Genome Research, 20(9), 1297–1303. Available from: 10.1101/gr.107524.110 20644199 PMC2928508

[age70055-bib-0027] Metzger, J. , Wöhlke, A. , Mischke, R. , Hoffmann, A. , Hewicker‐Trautwein, M. , Küch, E.M. et al. (2015) A novel SLC27A4 splice acceptor site mutation in Great Danes with ichthyosis. PLoS One, 10(10), e0141514. Available from: 10.1371/journal.pone.0141514 26506231 PMC4624637

[age70055-bib-0028] Mirdita, M. , Schütze, K. , Moriwaki, Y. , Heo, L. , Ovchinnikov, S. & Steinegger, M. (2022) ColabFold: making protein folding accessible to all. Nature Methods, 19(6), 679–682. Available from: 10.1038/s41592-022-01488-1 35637307 PMC9184281

[age70055-bib-0029] Muñoz‐Garcia, A. , Thomas, C.P. , Keeney, D.S. , Zheng, Y. & Brash, A.R. (2013) The importance of the lipoxygenase‐hepoxilin pathway in the mammalian epidermal barrier. Biochimica et Biophysica Acta, 1841(3), 401. Available from: 10.1016/j.bbalip.2013.08.020 24021977 PMC4116325

[age70055-bib-0030] Oji, V. , Tadini, G. , Akiyama, M. , Blanchet, B.C. , Bodemer, C. , Bourrat, E. et al. (2010) Revised nomenclature and classification of inherited ichthyoses: results of the first ichthyosis consensus conference in Sorèze 2009. Journal of the American Academy of Dermatology, 63(4), 607–641. Available from: 10.1016/j.jaad.2009.11.020 20643494

[age70055-bib-0031] Pejaver, V. , Urresti, J. , Lugo‐Martinez, J. , Pagel, K.A. , Lin, G.N. , Nam, H.J. et al. (2020) Inferring the molecular and phenotypic impact of amino acid variants with MutPred2. Nature Communications, 11(1), 1–13. Available from: 10.1038/s41467-020-19669-x PMC7680112 33219223

[age70055-bib-0032] Richards, S. , Aziz, N. , Bale, S. , Bick, D. , Das, S. , Gastier‐Foster, J. et al. (2015) Standards and guidelines for the interpretation of sequence variants: a joint consensus recommendation of the American College of Medical Genetics and Genomics and the Association for Molecular Pathology. Genetics in Medicine, 17(5), 405–424. Available from: 10.1038/gim.2015.30 25741868 PMC4544753

[age70055-bib-0033] Robinson, J.T. , Thorvaldsdóttir, H. , Winckler, W. , Guttman, M. , Lander, E.S. , Getz, G. et al. (2011) Integrative genomics viewer. Nature Biotechnology, 29(1), 24–26. Available from: 10.1038/nbt.1754 PMC3346182 21221095

[age70055-bib-0034] Takeichi, T. , Okuno, Y. , Saito, C. , Kojima, D. , Kono, M. , Morita, A. et al. (2017) Congenital ichthyosis and recurrent eczema associated with a novel ALOXE3 mutation. Acta Dermato‐Venereologica, 97(4), 532–533. Available from: 10.2340/00015555-2549 27868147

[age70055-bib-0035] Uitto, J. , Youssefian, L. , Saeidian, A. & Vahidnezhad, H. (2020) Molecular genetics of keratinization disorders—what's new about ichthyosis. Acta Dermato‐Venereologica, 100(7), adv00095. Available from: 10.2340/00015555-3431 32147742 PMC9128965

[age70055-bib-0036] Zheng, Y. , Yin, H. , Boeglin, W.E. , Elias, P.M. , Crumrine, D. , Beier, D.R. et al. (2011) Lipoxygenases mediate the effect of essential fatty acid in skin barrier formation: a proposed role in releasing omega‐hydroxyceramide for construction of the corneocyte lipid envelope. Journal of Biological Chemistry, 286(27), 24046–24056. Available from: 10.1074/jbc.m111.251496 21558561 PMC3129186

[age70055-bib-0037] Zhuravlev, A. , Gavrilyuk, V. , Chen, X. , Aksenov, V. , Kuhn, H. & Ivanov, I. (2024) Structural and functional biology of mammalian ALOX isoforms with particular emphasis on enzyme dimerization and their allosteric properties. International Journal of Molecular Sciences, 25(22), 12058. Available from: 10.3390/ijms252212058 39596127 PMC11593649

